# Effect of Meloxicam on the Proliferation and Apoptosis of the Raji Cell Line: an In Vitro Study

**DOI:** 10.1155/2022/9579326

**Published:** 2022-07-06

**Authors:** Yolanda Kartika Asmarani, Supriatno, Tetiana Haniastuti

**Affiliations:** ^1^Master of Dental Sciences Study Program, Faculty of Dentistry, Universitas Gadjah Mada, Yogyakarta, Indonesia; ^2^Oral and Maxillofacial Surgery Department, Faculty of Dentistry, Institut Ilmu Kesehatan Bhakti Wiyata, Kediri, Indonesia; ^3^Oral Medicine Department, Faculty of Dentistry, Universitas Gadjah Mada, Yogyakarta, Indonesia; ^4^Oral Biology Department, Faculty of Dentistry, Universitas Gadjah Mada, Yogyakarta, Indonesia

## Abstract

Meloxicam, a nonsteroidal anti-inflammatory drug, inhibits the production of PGE2 by blocking Cox-2 activity. Specific inhibition of Cox-2 can be useful in cancer therapy by apoptosis stimulation. The objective of the research was to study the effect of meloxicam on the proliferation and apoptosis of Raji cell lines. Burkitt lymphoma (BL) cells (Raji ATCC CCL-86) were treated with various concentrations of meloxicam for 24 hours. The proliferation of the cells was evaluated by using an MTT assay. Cell apoptosis was assessed using flow cytometry, and SEM was performed to observe the morphological changes of the cells. Results showed that meloxicam affected Raji cell proliferation as well as cell apoptosis. The percentage of viable cells was decreased significantly after being treated with meloxicam (*p* < 0.05). Apoptotic cell percentage was higher in the groups treated with meloxicam compared to the control group (*p* < 0.05). SEM showed morphological changes in the Raji cells after treatment with meloxicam, showing apoptotic characteristics. These findings suggest that meloxicam has anticancer properties by inhibiting Raji cell proliferation and inducing Raji cell apoptosis in vitro. A combination of meloxicam with chemotherapy agents may improve the outcome of BL treatment.

## 1. Introduction

Inflammation is a body's protective mechanism in response to tissue damage caused by various detrimental stimuli; however, improper and aberrant activation leads to deleterious effects [1]. Chronic inflammation induces cellular events that can promote stages of carcinogenesis by triggering immune and stromal cells to secrete various proinflammatory and oncogenic mediators including cytokines, nitric oxide, growth factors, and chemokines. These mediators tend to activate molecular signaling cascades that have a pleiotropic effect on inflammation and cancer progression [1, 2].

Cyclooxygenase 2 (Cox-2) is an essential biosynthetic enzyme in prostaglandin synthesis. Unlike Cox-1, which is constitutively expressed and associated with tissue homeostasis, Cox-2 is recognized to increase tumorigenic potential by inducing cell proliferation and resistance to apoptosis [3]. Upregulation of Cox-2 and increased synthesis of prostaglandins are known to promote cell proliferation, angiogenesis, and tumor invasiveness, while inhibiting immune surveillance and apoptosis. Overexpression of Cox-2 has been confirmed in some malignancies, including esophagus, lung, pancreas, prostate, and mucous membranes of the head and neck [4]. An elevated Cox-2 level has been demonstrated in B cell lymphoma cell lines [5]. A study by Paydas [6] revealed that Cox-2 expression was shown in 60% of cases with B cell non-Hodgkin lymphoma and was associated with aggressive morphology. Moreover, Cox-2 overexpression was related to resistance to apoptosis [7].

Burkitt lymphoma (BL) is a malignant non-Hodgkin B-cell lymphoma that originates in germinal center B cells. It is one of the most fast-growing malignancies in the human body [8]. Burkitt lymphoma involving the jaws is characterized clinically by displaced teeth, severely mobile teeth, and generalized lymphadenopathy [9]. Sariban et al. [10] reported that toothache and perioral numbness were the most commonly reported symptoms in adult patients, whereas loose teeth, toothache, and intraoral, as well as extraoral swelling, were the most frequent findings in children. Although the prognosis was very poor in the past, approximately 50–80 percent of adult patients with BL can now be treated with complex chemotherapeutic medication [11].

Cyclooxygenase 2 is a pivotal cellular target for therapy in malignancies. Specific blocking of Cox-2 can be useful in cancer treatment by apoptosis stimulation [12]. Numerous studies have reported that meloxicam, a Cox-2 selective inhibitor, impedes the proliferation of a number of cancer cell lines, such as ovarian [13], colorectal [14], hepatocellular [15], glioma cell [16], and osteosarcoma [17]. Research by Hazar et al. [18] reported that Cox-2 expression correlated clinically with prognostic factors in lymphoma patients.

Meloxicam, a derivative of enolic acid, is one of the commonly used nonsteroidal anti-inflammatory drugs (NSAID). Meloxicam preferentially suppresses Cox-2 activity more than Cox-1 [19]. The benefit of the Cox-2 selective inhibitor is its ability to decrease inflammatory prostaglandin synthesis without decreasing prostaglandin synthesis produced by Cox-1, which is essential in other body functions [20]. Previous studies by Goldman et al. [14] revealed that meloxicam affected the growth rate of HCA-7 cells (colon cancer cell lines that express Cox-2) by significantly reducing the colony size. The study also demonstrated that meloxicam inhibited the growth of HCA-7 xenografts in nude mice effectively.

Apoptosis, a physiological programmed cell death, occurs normally in many cellular systems [21]. Suppression of apoptosis or its resistance plays an important role in carcinogenesis. Hence, apoptosis is a promising target for anticancer therapy [22]. Researchers attempt to seek new drugs targeting apoptosis. Several in vitro studies have been carried out to explore chemotherapeutic drugs that can stimulate apoptosis in malignant cells [23, 24].

Considering that Cox-2 is a promising target for therapy in malignancies by apoptosis stimulation, this research aimed to study the effect of meloxicam on the proliferation and apoptosis of the BL cell line Raji.

## 2. Materials and Methods

### 2.1. Raji Cell Culture

The Burkitt lymphoma cell line Raji (ATCC CCL-86) was obtained from Parasitology Laboratory, Faculty of Medicine, Public Health and Nursing, Universitas Gadjah Mada, Indonesia. The cells were cultured in the RPMI-1640 medium (Sigma-Aldrich, St. Louis, MO, USA) supplemented with 10% fetal bovine serum, 100 IU/ml penicillin, and 10 g/ml streptomycin at 37°C with 5% CO_2_ content. The cells were subcultured for the study after 80% confluence was reached.

### 2.2. MTT Assay

To evaluate the proliferation of the Raji cells induced by meloxicam, tests were performed using the MTT assay method [25]. In brief, the cells were seeded in 96 well plates (1 × 10^4^ cells/well) and incubated at 37^o^C with 5% CO_2_ in the air. After overnight culture, the cells were incubated with various concentrations (0, 6.25, 12.5, 25, 50, 100, and 200 *μ*M) of meloxicam (Dexa Medica, Indonesia) at 37°C under a constant flow of 5% CO_2_. The viability of the cells was assessed 24 hours after treatment using the MTT assay. After the medium was discharged, MTT solution (200 *μ*l) was added to the wells and incubated at 37°C with 5% CO_2_ for 4 hours. Following the incubation, 10% sodium dodecyl sulfate (100 *μ*l) was added to dissolve purple crystals of formazan. Absorbance was measured in a spectrophotometer at a wavelength of 595 nm. Cell viability (%) was calculated as a percentage of absorbance relative to the untreated cells.

(1) $$\begin{array}{c} \%\text{ cell proliferation}=\frac{\text{OD treated group}}{\text{OD untreated group}}\times 100\%. \end{array}$$

### 2.3. Apoptosis Assay

Apoptosis was tested using the flow cytometry method [26]. The assay was carried out using the FITC-annexin V apoptosis detection kit (BD Biosciences) according to the manufacturer's instructions. Raji cells were seeded in 6 well plates at a density of approximately 2 × 10^4^ cells/well and treated with meloxicam at concentrations 0, 100, and 200 *μ*M at 37°C with 5% CO_2_ content. After being incubated for 24 hours, the cells were harvested and incubated with FITC-annexin V and PI for 15 min at 25°C in the dark. Flow cytometry was carried out within 1 hour by a FACScan flow cytometer (BD FACSCANTO II).

### 2.4. Scanning Electron Microscopy (SEM)

Morphology of the Raji cells induced by meloxicam was observed using SEM. The specimens were made 24 hours following cell treatment with meloxicam. After washing with PBS, the cells were fixed with 2.5% glutaraldhyde (Sigma-Aldrich, St Louis, MO, USA) for 2 hours at −18°C and seeded on a sample holder. The sample was then dehydrated through a graded acetone series and dried in a *vacuum system* (Buehler Cast n'Vac 1000, USA). Specimens were coated with gold (JEOL 780174712) in an auto fine coater (JEOL JEC-3000FC, USA) and observed with a JSM-6510LA SEM.

### 2.5. Statistical Analysis

Cell viability and apoptosis assays were repeated three times, and the data were expressed as the mean ± standard deviation (SD) from a representative experiment. Differences in cell viability and apoptosis, as well as necrosis, were analyzed using one-way ANOVA followed by a post hoc LSD test. Statistical analysis was performed using the Statistical Package for the Social Sciences, version 16.0. A *p* value lower than 0.05 was considered significant.

## 3. Results

### 3.1. MTT

**Figure 1:**
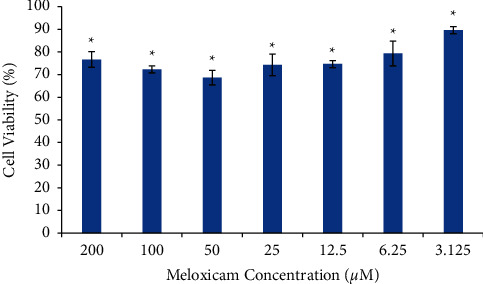
Raji cell viability was treated with various concentrations of meloxicam for 24 hours. A decrease in the viability of the cell as a response to meloxicam is shown. ^*∗*^*p* < 0.05 versus Raji cells untreated (control).

We examined the effects of meloxicam on the viability of Raji cells by using the MTT assay. Raji cells were treated with meloxicam at the specified concentrations (0–200 *μ*M) for 24 hours. The percentage of the cell viability were averaged to 89.67 ± 1.53%, 79.33 ± 5.51%, 74.67 ± 1.53%, 74.33 ± 4.73%, 68.67 ± 3.22%, 72.33 ± 1.53%, and 76.67 ± 3.51%, respectively. As shown in Figure 1, a significant decrease in cell viability was observed in Raji cells treated with meloxicam (*p* < 0.05). Although cell viability was slightly increased after being treated with 100 and 200 *μ*M meloxicam, the LSD test showed no significant differences that were found between both concentrations when compared with 50 *μ*M meloxicam (*p* > 0.05). All treated cells were significantly different compared to the untreated cells (*p* < 0.05).

### 3.2. Flow Cytometry

**Figure 2:**
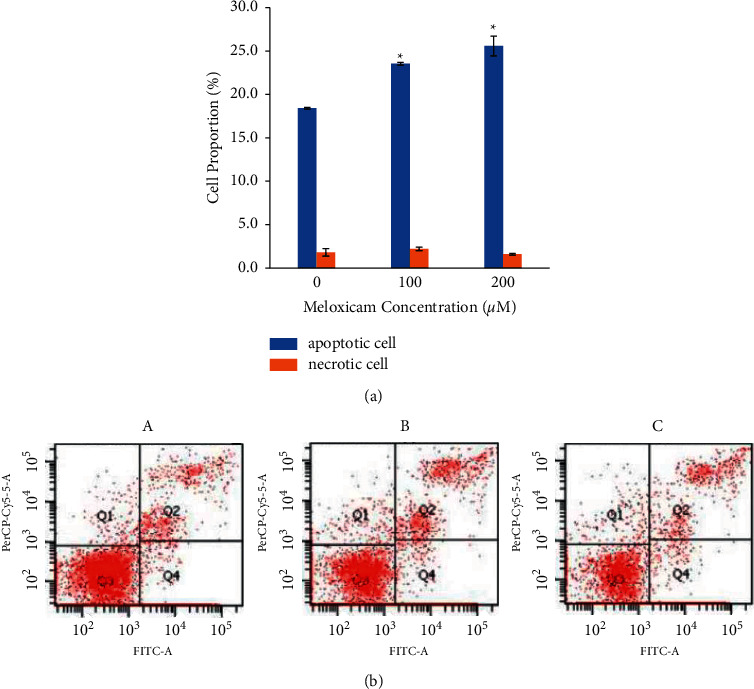
(a) The flow cytometry histogram of the percentage of Raji cells after 24 hours of incubation with various concentrations of meloxicam. ^*∗*^*p* < 0.05 versus Raji cells untreated (control). (b) Representative dot plots of Raji cells treated with meloxicam (untreated (A); 100 *μ*M meloxicam (B); 200 *μ*M meloxicam (C)). The percentage of apoptotic cells increased significantly after being treated with meloxicam (*p* < 0.05).

The apoptosis and necrosis of Raji cells were examined with flow cytometry at 24 hours of treatment with meloxicam.  Figure 2 shows that meloxicam treatment for 24 hours causes an increase in Raji cell apoptosis in a dose-dependent manner (*p* < 0.05). The percentage of apoptotic cells was averaged at 18.4 ± 1.8%, 23.53 ± 2.2%, and 25.57 ± 1.6%, respectively. Moreover, there was no significant difference in necrotic cell percentage among groups, indicating that meloxicam did not affect necrosis in Raji cells (*p* > 0.05).

### 3.3. Scanning Electron Microscopy

**Figure 3:**
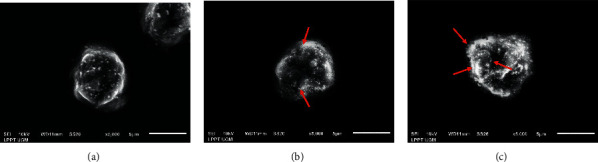
Representative scanning electron microscopy of Raji cells of control ((a) untreated) showing normal Raji cell characteristics and cells treated with meloxicam ((b):100 *μ*M; (c):200 *μ*M) showing the telltale morphology of an apoptotic cell with a ruffled and blebbed membrane.

Morphological changes in the Raji cells after being treated with meloxicam were observed using SEM (Figure 3). In normal cells, a rough surface with small microvilli was observed. After treatment with meloxicam, many blebs extrude from the cytoplasm giving the cell a foamy shape, indicating an apoptosis process occurred.

## 4. Discussion

Understanding the effects of certain substances on the body is essential for researchers and clinicians to determine the most appropriate therapy. Many in vitro and in vivo studies have been carried out to determine the response of cells, tissues, as well as the body's environment to a material [27–30].

BL is a highly aggressive B cell non-Hodgkin lymphoma characterized by a high proliferation rate of the cancer cells that can be fatal if not treated promptly [31]. The purpose of this research was to study the effect of meloxicam on the proliferation and apoptosis of BL Raji cell lines.

The molecular characteristic of BL is the activation of the c-Myc oncogene via reciprocal chromosomal translocations juxtaposing the c-Myc gene to one of the immunoglobulin loci [32]. Normally, the c-Myc protein regulates proliferation and cell survival, but when abnormally expressed, it increases cell cycle progression [33]. In addition, a majority of BL-derived cell lines carry point mutations in the p53 tumor suppressor gene or other defects in the p14ARF-MDM2-p53 pathway or inactivation of p16INK4a genes by promoter methylation or homozygous deletion. Thus, multiple genetic events that stimulate cellular proliferation and inhibit apoptosis are likely to be involved in BL [32, 34]. The ability to elude apoptosis is crucial for the development of lymphoma. Alterations in the principal regulators of apoptosis, the Bcl-2 family of proteins, are a characteristic of B cell lymphoma. Particularly, increased expression of antiapoptotic Bcl-2 family members and/or reduced expression of specific proapoptotic members are typical features of B cell lymphomas [35]. Pervez et al. [36] demonstrated strong diffuse cytoplasmic staining for Bcl-2 in specimens taken from an adult patient diagnosed with BL.

The proliferation of the Raji cells after being treated with meloxicam in this study was measured with the MTT assay. This assay is commonly used to assess cellular metabolic activity as an indicator of cell viability as well as proliferation through mitochondrial activity [37]. In this study, the MTT assay was performed after Raji cells were incubated with meloxicam for 24 hours [27, 28]. The result showed a significant reduction in Raji cell viability after being treated with meloxicam for 24 hours. This finding is supported by a previous report by Onen et al. [4] that meloxicam had an antiproliferative effect on Raji cells after a 24-hour incubation period. However, a study conducted by Kobayashi et al. [38] showed no significantly decreased cell viability of Raji cells treated with meloxicam for 72 hours. Studying cytotoxic effects of certain materials generally needs a 24-hour incubation period; however, an extended time of observation may provide better information for the researcher as well as the clinician [39]. Further research with extended observation periods is thus needed.

Several previous studies also demonstrated a statistically strongly suppressed proliferation of several cancer cell lines after treatment with meloxicam. Meloxicam was reported to have antiproliferative effects on human nonsmall cell lung cancer cell lines (A549 and PC14) and inhibit PGE2 production in these cells [40]. A study by Naruse et al. reported an antigrowth effect of meloxicam on human MG-63 osteosarcoma cells [41].

Meloxicam is a nonsteroidal anti-inflammatory drug that blocks PGE2 production by inhibiting Cox-2 activity [19]. The possible mechanism by which meloxicam inhibits cell proliferation is by modulating some cell cycle checkpoints. Meloxicam suppresses HepG2 cell line proliferation in a dose- and time-dependent manner, resulting in cell cycle arrest in S phase and cell accumulation in G0/G1 phase. The study also reported that the expression of cyclin A and PCNA in the HepG2 cell line was downregulated by meloxicam [15].

In addition to its ability to inhibit cell proliferation, flow cytometry analysis revealed that meloxicam enhanced Raji cell apoptosis. Furthermore, these findings were supported by SEM results. Taken together, the results are in accordance with the previous study by Li et al. [15] that meloxicam suppressed the growth and induced apoptosis of the HepG2 cell line.

Apoptosis is characterized by distinct morphological characteristics, namely, shrinkage of the cell, condensation of chromatin, fragmentation into membrane-bound apoptotic bodies, and membrane blebbing [42]. We found that after Raji cells had been treated with meloxicam, typical morphological changes of cell apoptosis were observed. After having been treated with meloxicam, SEM exhibited cells with a loss of surface microvilli and formation of membrane blebs. These phenomena showed a typical feature of cells undergoing apoptosis.

Based on the flow cytometry analysis and SEM observation, the present study showed that meloxicam induced cell apoptosis. However, the mechanism underlying meloxicam-induced apoptosis was unclear. The possible mechanism by which meloxicam induces apoptosis may be through Cox-2-dependent and -independent pathways. A study by Dong et al. [43] using the hepatocellular carcinoma (HCC) cell line demonstrated that meloxicam executed its anticancer effects by aiming at the Cox-2/MMP-2/E-cadherin, AKT, apoptotic, and autophagic pathways in Cox-2-dependent and Cox-2-independent pathways. They also showed that cell autophagy inhibition could assist to overcome the resistance to meloxicam-induced apoptosis in HCC. Meloxicam regulates apoptosis-associated proteins, surviving, as well as Mcl-1, via stimulation of AKT by inhibiting the production of PGE2, as PGE2 binds to its receptor (EP2) effectively, and in turn, it stimulates the AKT pathway. By inhibiting PGE2 production, meloxicam downregulates MMP-2 expression, which in turn increases E-cadherin expression [44]. Overexpression of E-cadherin impedes the pathway of PI3K/AKT via HER/IGF-1R [45]. In a COX-2-independent way, meloxicam stimulates apoptosis of the cell by upregulating Bax and Fas-L. In turn, 3-MA inhibits the engulfing of Bax by the autophagosome, hence blocking the inhibitory effect of autophagy on apoptosis [43].

In conclusion, this study provides evidence that meloxicam may have anticancer properties by inhibiting Raji cell proliferation and inducing cell apoptosis in vitro. The results of the present study support other studies investigating the possible efficacy of meloxicam against cancer cells. Furthermore, the results can be used to improve chemotherapy efficiency. However, the anticancer mechanism of meloxicam, especially the molecular signaling pathways involved in its anticancer effects, needs to be elucidated. In vitro and in vivo research are further required to determine the effects of meloxicam in combination with chemotherapeutic agents.

## Data Availability

The experimental data used to support the findings of this study are included within the article.
